# Genetic structure characterization of Chileans reflects historical immigration patterns

**DOI:** 10.1038/ncomms7472

**Published:** 2015-03-17

**Authors:** Susana Eyheramendy, Felipe I. Martinez, Federico Manevy, Cecilia Vial, Gabriela M. Repetto

**Affiliations:** 1 Department of Statistics, Facultad de Matemáticas, Pontificia Universidad Católica de Chile, Av. Vicuña Mackenna 4860, Macul, Santiago 6904411, Chile; 2 Interdisciplinary Center for Intercultural and Indigenous Studies, Anthropology Program, Institute of Sociology, Pontificia Universidad Católica de Chile, Av. Vicuña Mackenna 4860, Macul, Santiago 6904411, Chile; 3 Center for Genetics and Genomics, Facultad de Medicina, Clinica Alemana, Universidad del Desarrollo, Av. Las Condes 12438, Lo Barnechea, Santiago 7710162, Chile; 4 Hantavirus Program, Facultad de Medicina, Clinica Alemana Universidad del Desarrollo, Las Condes 12438, Lo Barnechea, Santiago 7710162, Chile

## Abstract

Identifying the ancestral components of genomes of admixed individuals helps uncovering the genetic basis of diseases and understanding the demographic history of populations. We estimate local ancestry on 313 Chileans and assess the contribution from three continental populations. The distribution of ancestry block-length suggests an average admixing time around 10 generations ago. Sex-chromosome analyses confirm imbalanced contribution of European men and Native-American women. Previously known genes under selection contain SNPs showing large difference in allele frequencies. Furthermore, we show that assessing ancestry is harder at SNPs with higher recombination rates and easier at SNPs with large difference in allele frequencies at the ancestral populations. Two observations, that African ancestry proportions systematically decrease from North to South, and that European ancestry proportions are highest in central regions, show that the genetic structure of Chileans is under the influence of a diffusion process leading to an ancestry gradient related to geography.

## Abstract (web summary)

Chileans are genetically admixed. Here, the authors find that the average admixing time is around 10 generations ago and show the contribution of European men and Native-American women to the Chilean population.

## Graphical abstract

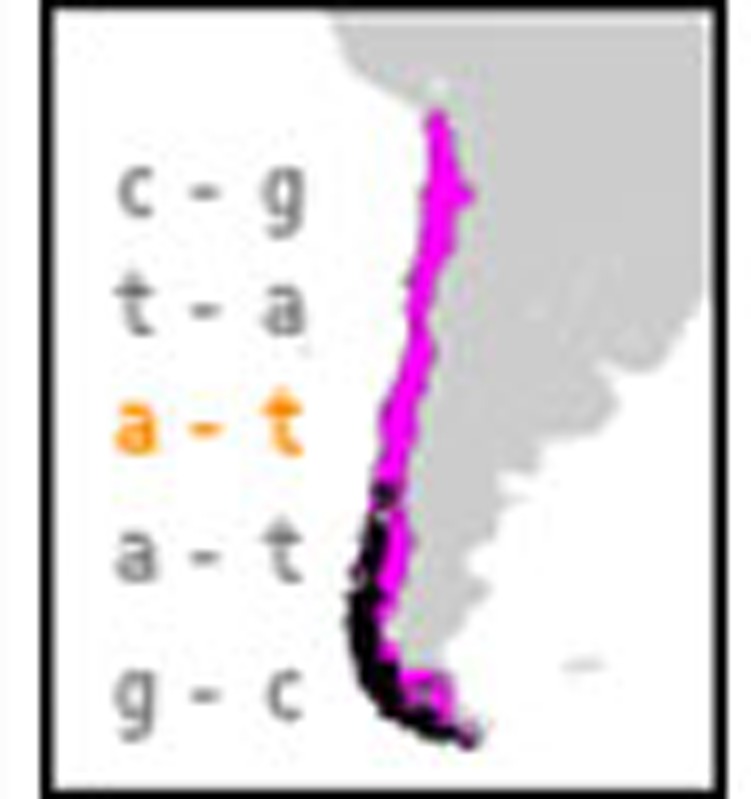

During the last decade vast advances in genotyping and sequencing technologies have led to better understanding of human genetic variation and its relation with natural selection, recombination rates and phenotype–genotype associations^1^,^2^,^3^,^4^. In particular, genome-wide association studies (GWAS) have shed light into numerous associations between diseases and genotypes^5^,^6^.

GWAS has worked well for homogeneous populations like Europeans. Unfortunately, for many populations that have recent ancestry from two or more continents (so-called admixed populations), like Latino and African Americans, this procedure can produce many spurious associations. A case–control design performed on an admixed population can produce spurious association when subjects in the case and control groups differ in allele frequency. This can easily arise from differences in ancestry proportions. It is therefore crucial to correct for ancestry to avoid false associations^7^,^8^,^9^,^10^,^11^.

Nevertheless, performing genome-wide association on admixed populations offers many advantages. First, recently admixed populations are likely to have a larger number of genetic variants with functional effects, as has been suggested by studies of natural selection^3^. Second, causal risk alleles can be searched by performing the usual comparison in allele frequency between cases and controls (correcting for population structure^7^,^8^,^9^,^10^,^11^), by performing an analysis of cases-only (admixture mapping^12^), or by using cases-and-controls to search for unusual deviation in local ancestry. SNP and admixture association signals contain information that can complement each other^13^, and more powerful statistical tests that combine the two signals can be applied^8^,^14^.

The Latino-admixed populations offer particular challenges for performing GWAS, for example, it is difficult to choose accurate ancestral populations for Latinos^15^,^16^, Native-American panels are still sparse and genetically heterogeneous and also existing methods for multi-way local-ancestry inferences (three or more ancestral populations), which are necessary for Latino populations, show biases in their miscalled segments^17^.

In this paper, we aim to increase our knowledge of the genetic patterns of the Chileans. The genetic composition of the Chileans is shaped by the interplay of ancient populations and recent demographic shifts. Chile is located along the south west coast of the Pacific ocean in South America, bordering on the east with one of the highest chain of mountains in the world, the Andes. Arguably, this isolation may have resulted in particular genomic traits, from the ancient ancestral populations, that are advantageous for genetic mapping. In contrast, the Chileans are admixed. The discovery and conquest of Chile by the Spaniards began in the mid-sixteenth century, starting a Caucasian-Native-American miscegenation during the last 16 generations^18^. In addition, during the seventeenth century, a minor African component was brought in. After the Spanish conquest, new migrations mainly from Europe occurred during nineteenth and twentieth centuries, which contributed to the development of modern Chileans.

The study of the genetic structure of the admixed Chileans can shed light on the genetic basis of diseases through more powerful tests, as mentioned above. In addition, genetic studies of minority populations are needed to develop truly global medical genomics^19^. However, to accurately perform association studies in this population, it is necessary to infer local ancestry, and therefore it is essential to assess local ancestry estimations performance in empirical data^20^. Local ancestry inference (LAI) consists on the assignment of a label of ancestral origin to each position of the genome. It has a wide range of applications from pharmacogenomics to human demographic history and natural selection^3^,^21^,^22^, and numerous computational approaches have been developed^17^,^23^,^24^,^25^,^26^,^27^. In general, LAI methods receive as input unphased genetic data from an admixed individual, and output the ancestral origin of each chromosomal segment based on a model and a reference panel of ancestral populations.

In this work, we characterize the patterns of genetic variation on the Chileans using genotype data on ~685,944 SNPs from 313 individuals across the whole-continental country, with the purpose of providing essential information to future medical genetic association studies. We identify SNPs with highly differentiating allele frequencies and estimate an average time since admixture that is around 10 generations ago. On the basis of LAI, we show that assessing ancestry is in general more difficult at SNPs with higher recombination rates and easier at SNPs that have a large difference in allele frequency at the ancestral populations. Also, African ancestry proportions systematically decrease from North to South and European ancestry proportions are highest in central regions.

We assess the sensitivity of local ancestry estimation methods on sample size of the ancestral populations, showing that the methods are robust. We also show that the errors in ancestry estimation can be large when the density of the SNPs in the panels is small. We further evaluate the consistency between two state-of-the-art codes used for LAI, showing sufficient consistency on global and local ancestry estimation.

## Results

### Analysis of population structure

**Figure 1:**
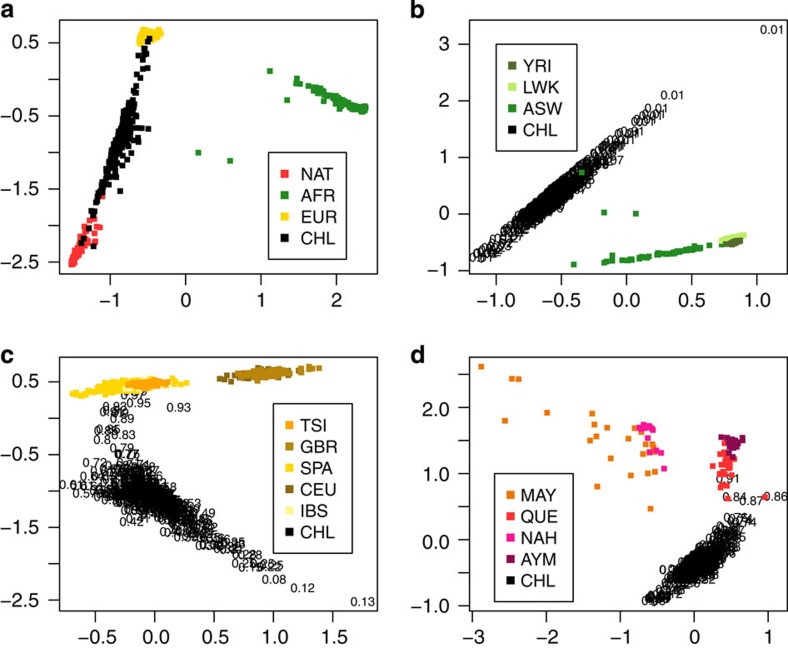
Spatial ancestry analysis. (**a**) All populations, (**b**) Chilean and African populations, (**c**) Chilean and European populations and (**d**) Chilean and Native-American populations. Chileans appear in black with its global African, European and Native-American ancestry proportions in (**b**), (**c**) and (**d**), respectively, as estimated by local ancestry methods.

We perform an analysis of spatial ancestry^28^, results are shown in Fig. 1. Figure 1a shows that the spatial analysis clearly separates the three ancestral population and, as expected, the admixed Chileans are scattered between the European and the Native-Americans. We restrict our spatial analysis to the Chileans with each of its ancestral populations. Figure 1b shows the Chilean-admixed population clearly separated from the African populations. Within Africans, the Yoruba in Ibadan, Nigeria (YRI) and the Luhya in Webuye, Kenya (LWK) are close together, but the African Ancestry from Southwest United States (ASW) is spread along a line and is the closest to the Chileans. Figure 1c shows that the Europeans are separated into two groups, Britain (GBR) and North Europe (CEU) together and Spaniards and Italians together, closer to the Chileans. Figure 1d shows the Chileans together with the Native-Americans. Figure 1b–d shows the Chileans with their global proportion of African, European and Native-American ancestry, respectively, estimated by the LAI method. We observe consistency between the ancestral proportion estimates given by the LAI methods and the spatial ancestry analysis as individuals with higher/lower proportion in ancestry are closer/farther to the ancestral populations.

### Ancestry informative SNPs

**Figure 2:**
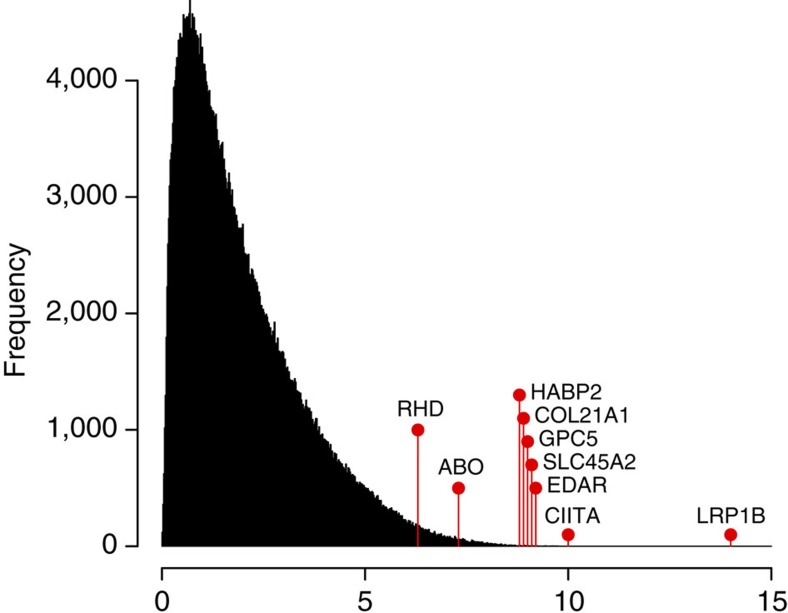
Distribution of scores obtained from the SPA software. Marked positions correspond to SNPs from genes discussed in the text.

Spatial analysis detects hundreds of SNPs with large frequency difference at Native-American (NAT), European (EUR) and Chilean (CHL) panels (see Supplementary Table 1 for a list of the top 100 loci). Some of these SNPs are located near or within genes known to have undergone recent positive selection or related to phenotypes. Examples of such informative genes are EDAR and SLC45A2 (see Fig. 2). Further analysis should be performed to identify whether any of these genes have undergone recent positive selection.

### Estimating ancestry proportions with LAI methods

We obtain local ancestry proportion estimates for each individual in the Chilean sample, at each of the 685,944 specific loci along the 22 pairs of autosomal chromosomes included in this study. We use haplotypes reference panels of sizes of 176, 410 and 458 individuals for the NAT, EUR and AFR ancestral population, respectively.

### Individuals’ ancestry

**Figure 3:**
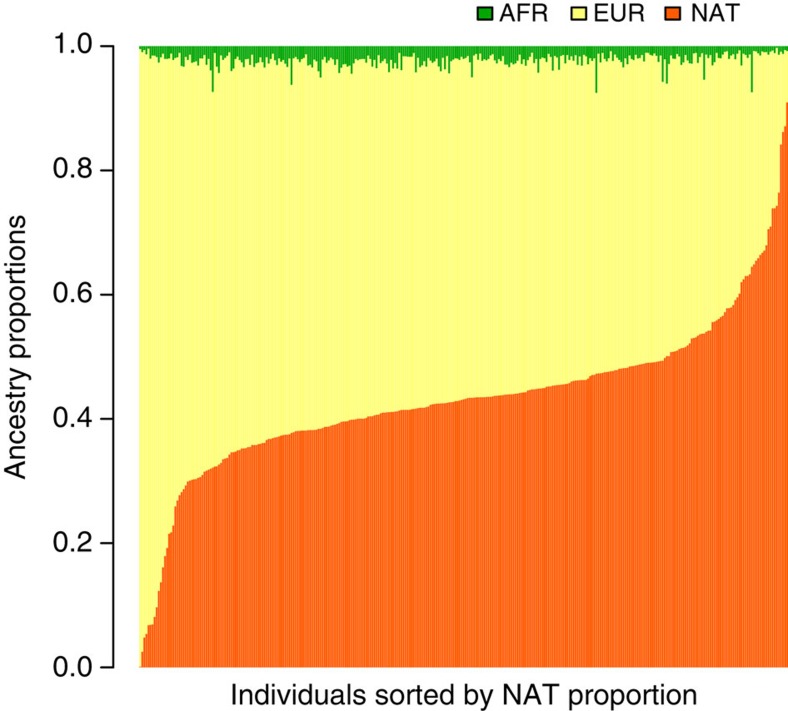
Individual global ancestry estimates from LAMP-LD. From left to right, Chilean samples are sorted in ascending order of Native-American ancestry proportion.

By summing up the local ancestry contribution inferred by LAMP-LD/RFMix, we obtain estimates of global ancestry proportions for each Chilean individual. Results from LAMP-LD are shown in Fig. 3. Each vertical bar represents an individual with the proportion of each ancestry depicted in a different colour. NAT ancestry percentages range from 0.13 to 90.95%, EUR ancestry percentages range from 8.26 to 99.41% and AFR ancestry percentages range from 0.26 to 7.50. According to the analyses performed on Error Evaluation, on average these global ancestry estimators should not deviate in more than (1.06*e*−04, 5.53*e*−05, 6.75*e*−07) at the (NAT, EUR, AFR) proportion, respectively, due to the size of the reference panels considered, and should not deviate in more than (1.5*e*−05, 1.8*e*−05, 1.68*e*−05) at the (NAT, EUR, AFR) proportion, respectively, due to the SNP density considered.

### Population ancestries

To estimate global Native-American, European and African ancestry proportions in the Chileans, we compute weighted averages of LAMP-LD’s global ancestry proportions across the 313 individuals. We find CHL ancestry percentages being 42.38% NAT, 55.16% EUR and 2.44% AFR (using LAMP-LD) and 43.22% NAT, 54.38% EUR and 2.40% AFR (using RFMix), which are consistent with previous studies^29^.

### Population ancestries at demographic zones

**Figure 4:**
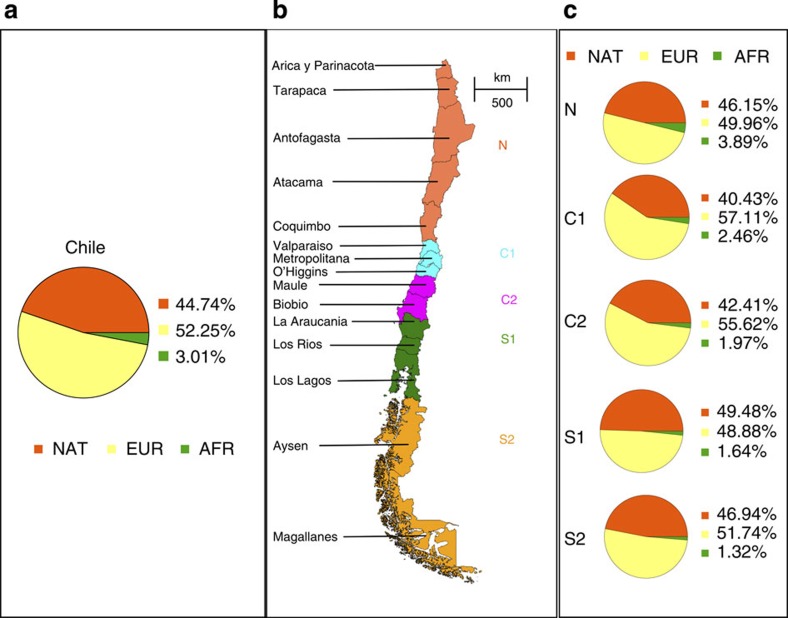
Global ancestry estimates per zones. (**a**) The larger pie chart on the left panel corresponds to global ancestry proportion for the Chileans as a whole; (**b**) the panel in the middle is a map of Chile describing the subdivision into five zones: N (*n*=16), C1 (*n*=159), C2 (*n*=41), S1 (*n*=73), S2 (*n*=12) and the political regions associated with each zone; (**c**) on the right panel, the pie charts correspond to global ancestry estimation on the five zones.

We estimate the ancestry proportions at every geographic zone (see Fig. 4) by averaging LAMP-LD’s global ancestry estimates of the individuals from each zone.

**Figure 5:**
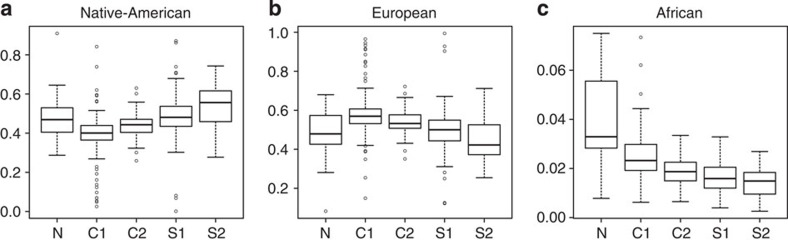
Global ancestry distribution per zones. The box plots represent the distribution of global ancestry proportions at every zone. The horizontal line inside the box corresponds to the median of this distribution, the bottom and top of the box are the first and third quartiles and data points outside the whiskers can be considered outliers. The sample sizes are 16, 159, 41, 73 and 12 for zones N, C1, C2, S1 and S2. respectively. Figure (**a**) shows the Native-American ancestry proportions, (**b**) the European proportions and (**c**) the African proportions over all the Chilean sample.

From Fig. 5a,b it can be observed that the central regions C1 and C2 have larger proportion of European ancestries (and smaller proportion of Native-American ancestry), which are statistically significantly different from the proportions at the remaining zones.

Further, the mean African ancestry percentage systematically decreases from North to South (see Fig. 5c), being 3.89% in the northernmost region N, 2.46% in the region C1, 1.97% in region C2, 1.64% in the region S1 and finally 1.32% in the southernmost region S2. The Tukey test for multiple comparisons shows that the means are significantly different between the zone N and C1 and between C1 and C2, but the means at C2, S1 and S2 are statistically equivalent. Arguably, larger sample sizes on these regions are needed to show a statistically significant decrease in African ancestry as we move to the southern regions of Chile.

### Gender imbalance

We calculate pairwise *F*_ST_ statistics and test for the null hypothesis that there is no difference between the populations. From pairwise *F*_ST_ estimates in chromosome Y, we confirm a predominant European ancestry, *F*_ST_=0.07 (*P* value<10^−5^) between Chileans and Europeans compared with an *F*_ST_=0.27 (*P* value<10^−5^) between Chileans and Native-Americans. From the analysis on chromosome X, we observed predominant Native-American ancestry *F*_ST_=0.0013 (*P* value<10^−5^) between the Chileans and the Europeans compared with an *F*_ST_=0 (*P* value<10^−5^) between Chileans and Native-Americans.

### Estimating time since admixture

**Figure 6:**
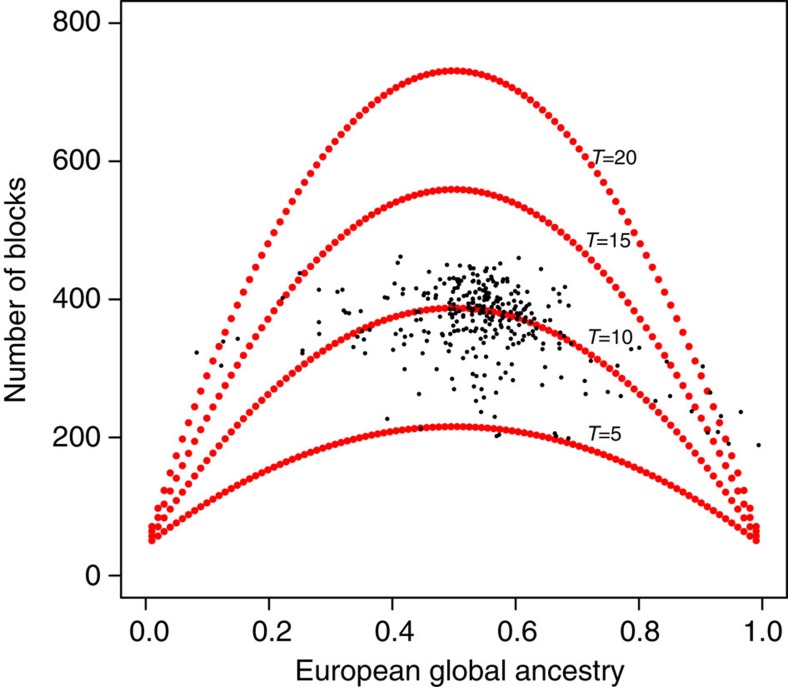
Estimating time since admixture by the number of ancestry blocks. Red dots represent the theoretical expected time (*T*) since admixture as a function of genome-wide European ancestry proportion. Black dots represent the Chilean sample data.

We count the number of ancestry blocks at each individual to estimate an average, *T*, time since admixture. We assume a hybrid-isolation model to estimate the expected number of ancestry switches from a population admixed *T* generations ago^22^. A more suitable, but mathematically more involved model, should assume continuous gene flow as well, so we need to interpret our results as an estimate of average admixing time weighted by the relative level of gene-flow per generation. Figure 6 shows the theoretical expected time since admixture for *T*ε{5, 10, 15, 20} generations ago as a function of genome-wide European ancestry proportion (red dots). Black dots represent the Chilean sample, which is scattered around *T*=10 generations ago, consistent with previous studies^30^.

### Comparison between LAMP-LD and RFMix in local ancestry

We evaluate the consistency between local ancestry estimates at each individual by calculating the percentage of coincidences in the local ancestry assignment of both methods (see Supplementary Table 2). Note that 82.35% of the ancestry assignments coincide exactly between the two methods. The largest differences occur at Native-American/European assignments by LAMP-LD/RFMix (8.31%) or inversely, EUR/NAT assignments by LAMP-LD/RFMix (7.33%). We further compare RFMix with and without EM iterations, and obtained a 1% improvement at the number of consistent assignments when the EM iterations were performed.

We evaluate the consistency between global ancestry estimates at each individual between both local-ancestry methods by counting the number of individuals, which differ in its global ancestry percentage in less than 0.1, 0.5, 1, 3, 6% (see Supplementary Table 3). Differences in Native-American ancestry estimates across methods are always<6% (with 88.5% of individuals always <1%), and this holds for differences in European ancestry estimates too (with 87.9% of individuals always <1%). Differences in African ancestry estimates are always<3% (with 90.4% individuals always <0.5%). Therefore, we observe substantial global ancestry consistency between the two methods.

We perform correlation tests to further evaluate whether SNPs with larger inconsistency between the methods correlate with recombination rates, or with SNPs with large frequency differences at the ancestral population. The Pearson correlation test that assesses the association between the number of inconsistencies between the methods and the recombination rate computed at each SNP has a *t*-statistics=100.86 with df=531,249 and a *P* value <e−16, which shows that the correlation is statistically significantly different from zero. In this test, we observe positive correlation, meaning that for lower recombination rates we should expect less inconsistencies between the methods, and more inconsistency when the recombination rates are higher. The correlation test that measures the association between the number of inconsistencies between the methods and the square difference of the frequencies at the Native-American and European population computed at each SNP has a *t*-statistics=−13.91 with df=531,249 and a *P* value <e−16, which also shows that the correlation is statistically significantly different from zero. In this case, we have negative correlation, meaning that at SNPs with larger frequency differences at the main ancestral populations we should expect a lower number of inconsistencies between the methods.

### Comparison of LAMP-LD and ADMIXTURE in global ancestry

**Figure 7:**
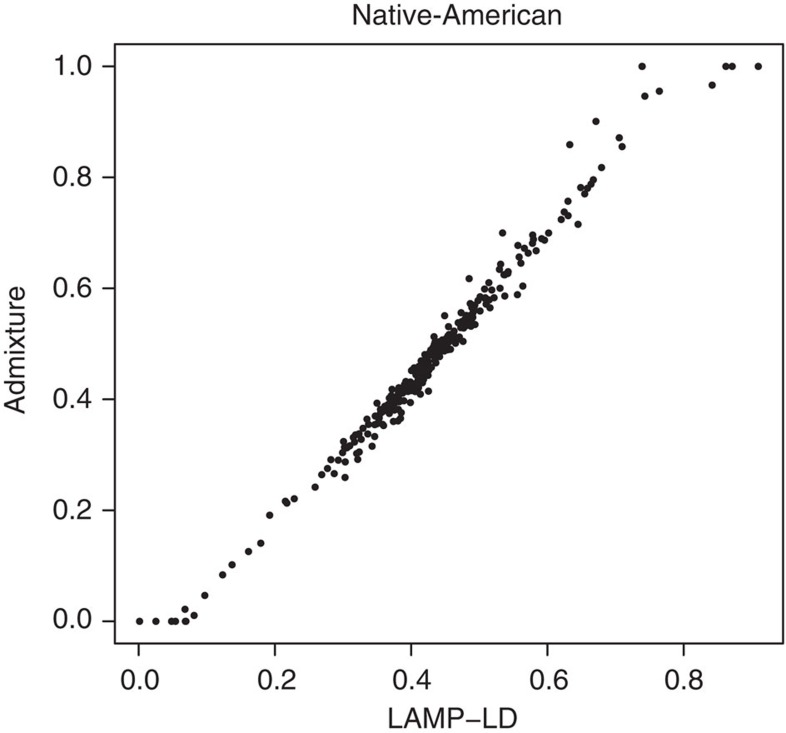
Comparison between NAT ancestries on LAMP-LD and ADMIXTURE. Black dots represent the global proportion of NAT ancestry as estimated by ADMIXTURE (*y*-axis) and LAMP-LD (*x*-axis) for each individual in the Chilean sample.

We compare global ancestry proportions at each individual between the global ancestry estimates obtained from LAMP-LD and ADMIXTURE^31^. We run ADMIXTURE with *K*=2 and *K*=3, number of ancestral populations, under the unsupervised mode. We obtain a correlation on the NAT ancestry equal to 0.99, for *K*=2, and 0.96 for *K*=3. Figure 7 shows the correlation on global ancestry estimates based on these two methods.

### LAI’s sensitivity to the size of the reference panels

We measure the global ancestry proportions, using LAMP-LD’s LAI, in the Chilean sample as we increase the size of each of the three reference panels simultaneously from 10 until 176 haplotypes. We estimate the variability on the global proportions estimate for each individual by repeating the procedure 10 times. Supplementary Figure 1 shows how the mean and s.d. of the Chilean global ancestry proportion estimates vary as a function of the size of the reference panels. We select *X*_0_=130 haplotypes per reference panels as the minimum starting size for the three ancestry panels.

**Figure 8:**
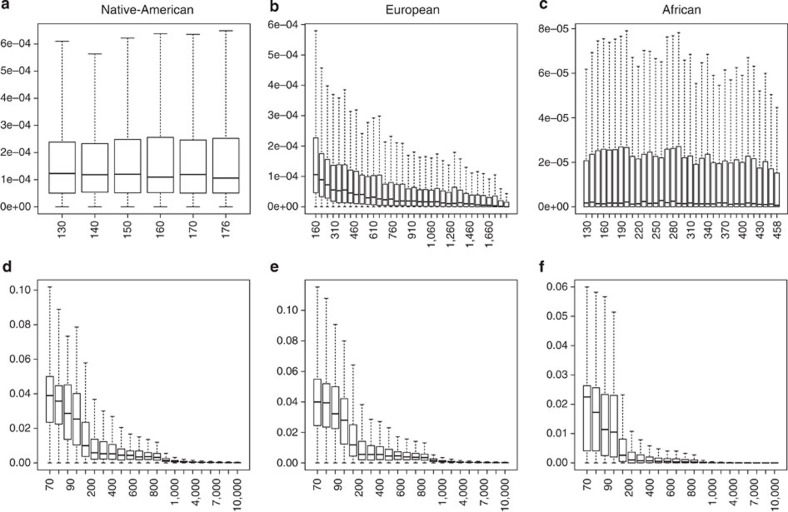
Standard error distributions. The box plots represent the distribution of the s.d. values over the Chilean sample (*n*=313).The first row estimates the distribution of s.d. values at different panel sample sizes: (**a**) Native-American, (**b**) European and (**c**) African, respectively. The second row shows the distribution of the sample errors over the Chilean sample at different SNP densities: (**d**) Native-American, (**e**) European and (**f**) African, respectively.

We start with *X*_0_=130 haplotypes per reference panel and increase the number of Native-American reference haplotypes. Native-American ancestry estimates increase from 42.23 to 43.03%, while European dropped from 55.45 to 54.72% and African dropped from 2.32 to 2.25%. Figure 8a shows the distribution of the s.d. of the estimates of Native-American proportion as we increase the ancestral reference panel for this population. This figure shows stable and similar distribution as we increase the size of the Native-American reference panel, at *X*_Am_=176 a slightly smaller median s.d. is achieved (median_sd_=1.06*e*−04). We fix *X*_Am_=176.

Increasing the size of the EUR reference panel caused an increase in the global estimates of EUR ancestry proportion, at the expense of both AFR and NAT ancestry estimates. We start with haplotype reference panels of size *X*_Am_=176 in NAT, 130 in EUR and 130 in AFR. We increase the number of EUR haplotypes in tens until the maximum number of 1822 haplotypes is reached. Estimates of ancestry percentages change from 43.03, 54.72 and 2.25% to 40.75, 57.84 and 1.41% in NAT, EUR, AFR, respectively. A combination of reference panel sizes such as the latter, where two sets are relatively small compared with the third one, might cause an overestimation of ancestry proportions related to the largest reference panel. We set the optimal number of EUR haplotypes to *X*_E_=410 for which we find a minimum median s.d. of median_sd_=5.53*e*−05. Figure 8b shows box plots of the distribution of the s.d. of the estimates of European ancestral proportion as we vary the size of this reference panel.

Increasing the size of the AFR reference panel causes an increase in the global estimates of AFR ancestry proportion, mainly at the expense of EUR ancestry estimates. We start with (NAT, EUR, AFR) reference panels of size (*X*_N_=176, *X*_E_=410, *X*_*0*_=130), and increase the number of haplotypes in the AFR reference panel. Estimates of ancestry percentages change from (41.22, 57.17 and 1.61%) to (41.17, 56.77 and 2.06%) for the total number of haplotypes. The small changes observed in the percentages of ancestry are expected as the Chilean population have small percentages of African ancestry. We set *x*_A_=458 at which it is reached a minimum median s.d. of median_sd_=6.75*e*−07. Figure 8c shows box plots of the distribution of the s.d. of the estimates of African ancestral proportion as we vary the size of this reference panel.

### SNP density effect on global ancestry estimation

We measure the s.d. on the global ancestry estimators of each individual by sampling randomly 10 times different sets of SNPs of the same sample size from chromosome 1. Figure 8d–f shows box plots of the distributions of the s.d. over the Chilean sample at each sample size and for each ancestral population. Note a rapid decay on the median s.d. as SNP density increases. For the Native-American ancestry, the median s.d. starts at 0.108 for a SNP density of 10 and ends at 1.5*e*−05 for a SNP density of 10,000. Similarly for the European ancestry, the median s.d. starts at 0.112 for a SNP density of 10 and ends at 1.8*e*−05 for a SNP density of 10,000. For the African ancestry, the median s.d. starts at 4.69*e*−02 for a SNP density of 10 and ends at 1.68*e*−05 for a SNP density of 10,000.

## Discussion

In this work, we study the genetic structure of the Chileans through spatial ancestry analysis and by estimating the local ancestry at every individual from the sample. Even though our sample was not selected for this type of study, it is representative of continental Chileans. There are no previous studies showing ancestry correlation to 22q11 microdeletion syndrome (the deletion covers less than 250 SNPs from the panel) or Hantavirus infection. We have individuals from all the regions of the country, and we include a correction for over-sampling southern regions and under-sampling northern regions.

From the spatial ancestry analysis, we confirm that Chileans are admixed with ancestral contribution mainly from Europe and Native America, with a minor African component. Within the Africans, the YRI and the LWK are close together but the ASW is spread along a line and is the closest to the Chileans, arguably because of the historical evidence corroborating a West African origin for the African lineages in the Americas. We also confirm that from the European components, the Chileans are closer to Spaniards and Italians than to British and CEU. This observation is in agreement with Chilean immigration history, as Chile was conquered by the Spaniards during the sixteenth century. From the Native-American components, we observe that the Natives from Peru and Bolivia (Aymara and Quechuas) are clustered together and are closer to the Chileans, as expected, than the North American natives (Mayas and Nahua), which are farther away and more spread. This suggests higher heterogeneity in Native American populations compared with Europeans or Africans, which is in agreement with the observation that obtaining optimal ancestral populations for Latin Americans is more difficult than for other admixed populations^17^.

Also from the spatial analysis, we identify interesting loci showing highly differentiating allele frequencies. Some of these loci are found within or nearby genes previously reported as candidate genes under selection. For example, *EDAR* has several SNPs with high SPA-score. Previous studies reported signals of recent positive selection and associate *EDAR* with hair morphology and sweat glands density in Asians and Native-Americans^32^,^33^,^34^,^35^,^36^. *SLC45A2* also shows SNPs with high SPA-score. This gene has been associated with skin and hair pigmentation, and several studies found signals of recent positive selection^32^,^33^,^34^,^35^,^36^,^37^. Our spatial analysis reaffirms that *SLC45A2* and *EDAR* hold clues to explain important aspects of the evolutionary history of Native-Americans. A large SPA-score is obtained in an intronic SNP from the *LRP1B* gene (also previously reported in ref. 28). This gene codifies a low-density lipoprotein receptor^38^,^39^ and has been related to body mass index in US and Danish individuals^40^,^41^. Other examples of informative genes identified are *CIITA, HABP2, COL21A1* and *GPC5*. *CIITA* encodes a transactivator of *MHC* class II^42^ and has been associated with susceptibility to numerous diseases including lymphoma^43^. *HABP2* codifies a serine protease with a role in coagulation/fibrinolysis-related activities, as well as an inflammatory mediator^44^. *GPC5* is related to inflammatory response and has been identified to be under selection favouring resistance to cholera disease^45^. *COL21A1* codifies the type *XXI* collagen, a component of extracellular matrix of blood vessel walls^46^. In addition to these genes, Fig. 2 highlights *RHD* and *ABO* genes (Rh and ABO blood group system, respectively). Both exhibit relatively high SPA-scores, which is expected for classic genetic markers widely assessed during last century, and frequently used in the Chilean and Latin American populations^47^,^48^,^49^.

Our study provides the first evaluation genome-wide of local ancestry on the Chileans and provides estimates of s.e. values of individuals’ global ancestry for different SNP densities and sizes of reference panels that can be used to design future GWAS in Latino-admixed population. Our results show that the distribution of errors is in general small as we vary the size of the reference panels. Caution should be taken when one of the ancestry panels is much larger than the other panels. In our case, the European panel is much larger than the African and Native-American panels, so increasing the European panel to its largest size may provoke an over estimation of European ancestry, mainly at the expense of Native-American ancestry. Our analysis also shows that the errors decrease substantially as we increase the SNP density, showing that for a small number of SNPs errors can be unacceptably high.

Good agreement is observed between the two LAI methods regarding individual global ancestry proportions and substantial agreement in local ancestry estimates. Previous studies have shown that loci with increased deviation in LAI show increased Mendelian inconsistency rates in LAI in Latinos^20^. Our study shows that loci with higher rates of inconsistency between LAMP-LD and RFMix have negative correlation with loci with increased deviation in frequency at the ancestral populations, this is expected, as SNPs with larger deviation in allele frequency at the ancestral population should be more informative for ancestry inference. Also, our study shows that loci with higher rates of inconsistency between the two LAI methods have positive correlation with higher recombination rates. At higher recombination rate loci, the methods should decide whether to switch from the haplotype from which they have been copying, and if a switch is performed, whether they switch to a haplotype from the same ancestral population or to another ancestral population. Therefore, there is more uncertainty on what is the optimal solution and is then natural that the methods have a higher rate of inconsistency at such loci with higher recombination rates.

Our results reflect the demographic history of post-Columbian colonization of Chile. First, our results from sex-chromosome analyses confirm the imbalanced contribution of European men and Native-American women to the Chileans, reported by previous studies of mitochondrial and nuclear DNA markers^50^,^51^. Second, the observation that African ancestry proportions systematically decrease from North to South is in agreement with known local history of African slavery brought to the Pacific shore of South America. The northern regions of Chile received an important number of African slaves during the Colonial period^52^,^53^. However, this was not the case for the central and southern regions. While a great number of African slaves were brought to Peru to work on plantations and mines, Chile received a low number because of its small economic activity. This explains the decrease in African ancestry proportion from North to South, as a gradient dependent on proximity.

Third, the observation that European/Native-Americans ancestry proportions are the highest/lowest in central regions C1 and C2 reflects the historical concentration of inhabitants in the centre of Chile. Now-a-days, the territories grouped in this study as central regions C1 and C2 concentrate 75% of the total population of Chile. This demographical fact means that central regions received much higher numbers of immigrants than other zones. The causes of this can be traced to several historical factors. During Colonial times, Chile occupied a more reduced territory than today. The North corresponded to a different political government and its boundary area, called Atacama, was usually referred as *despoblado* or unpopulated. In the South, Mapuches, the only indigenous group that was not subjugated by Spanish conquerors, maintained a military frontier south to the Biobío River (which coincides with the C2/S1 region demarcation used in this study). The entrance and occupation of non-indigenous population to these territories occurred after 1860, when Chile was an independent republic^54^. In addition to this, the extreme south of Chile (S2 region) was only recently populated. Consequently, most of the northern and southern regions of Chile were populated in recent times. Despite the several waves of European migrants that were moved to colonize extreme northern and southern areas, the central regions consistently kept the majority of this immigration during the last two hundred years.

The correlation between geography and genetic diversity has been widely recognized. Earlier studies show that allele frequencies follow an increasing/decreasing gradient pattern that extend over the entire world^55^,^56^. While the regional structure of admixture in the Chilean populations seems homogeneous compared with other countries of Latin America^15^, the north to south changes in allele frequency for African and from centre to the periphery for European and Native American ancestry show that the spreading of these alleles is dependent on geography. This pattern has been recently observed at lower resolution of markers^57^.

Overall, the results from global ancestry estimates per zones show that the genetic structure of Chileans is not uniformly distributed along the country. Both observations (African ancestry proportions systematically decrease from North to South, and the highest in central regions C1 and C2) suggest that, at the micro-evolutionary scale, the genetic structure of the Chileans is under the influence of a diffusion process leading to a gradient pattern of variation related to geography. Further analyses including additional data points are needed to corroborate this gradient pattern of variation.

## Methods

### Sample collection and genotyping

We estimate local ancestry on 313 Chilean admixed samples from two case–control studies, on Hantavirus infection (*n*=112) and 22q11 microdeletion syndrome (*n*=201), genotyped on an Affymetrix 6.0 GeneChip Array (Santa Clara, CA). Table 2 shows the distribution of the samples across the 15 political regions in Chile. Local Ethics Committees at each participating institution approved the study and informed consent was obtained from all participants, their parents or legal guardians. Local ancestry estimation requires reference panels from the ancestral populations, which for latinos correspond to Native-American, European and African populations. Our reference panels consist of 88 Native-Americans, 911 European and 229 Africans (Table 1 shows details on the populations). We filter SNPs for minor allele frequency (>0.01), Hardy–Weinberg Equilibrium (*P* value>10^−5^), call rates (>95%), missingness on SNPs and samples (10%) and discard A/T and C/G SNPs. We further filter out SNPs from the HLA region to avoid bias in the estimation of local ancestry^58^. A total of 685,944 SNPs remain. Phase data are obtained using BEAGLE^59^ on the CHL, NAT and SPN populations. To provide further demographic insights we grouped the Chilean sample into five zones from North to South (N,C1,C2,S1,S2) as shown in Fig. 4.

### Analysis of population structure

A cluster analysis is performed using the SPA software^28^ to identify the spatial structure in two dimensions of the Chilean sample with its ancestral populations. The unsupervised SPA analyses performed under default parameters estimate the allele frequency at every SNP as a function of geographical position. SNPs with large differences in allele frequency at the populations studied are more informative to distinguish individuals with different ancestries and are among candidate loci under selection.

To determine gender imbalance in ancestry, we calculate Slatkin’s *F*_*ST*_ values between Native-Americans, European and Chileans, using chromosome X on female samples and chromosome Y on male samples.

### Local ancestry inference

We infer local ancestry at the Chilean sample using LAMP-LD^17^ and RFMix^27^ softwares. Results from RFMix are obtained without the EM iteration option. We evaluate the sensitivity of these softwares on the size of the reference panels, and the density of SNPs, and we evaluate the consistency between the two methods. We compute global ancestry proportions at each ancestral population for every individual from the Chilean sample by calculating the proportion of bases assigned to each ancestral population. From the ancestral proportions at each individual we estimate the ancestral proportions at the admixed Chileans. From Table 2 we can note that the regions in the north are in general under-sampled and the regions in the south are in general over-sampled. Therefore, to obtain unbiased estimators of the population global ancestry proportions for Chileans and at the five demographic zones (Fig. 4), we calculate weighted averages over all the individuals in the sample. We determine a single weight for every region as the ratio between the population and sample fractions for that region, and every individual from the same region shares the same weight. For example, for ‘Arica y Parinacota’ the weight is calculated as w=1.1/0.6=1.83 while the weight for region ‘Metropolitana’ is w=40.3/39.6=1.02. If *p*_*i*,NAT_, *p*_*i*,EUR_ and *p*_*i*,AFR_ correspond to the Native-American, European and African ancestry proportion, respectively, estimated at individual *i* from the sample, then


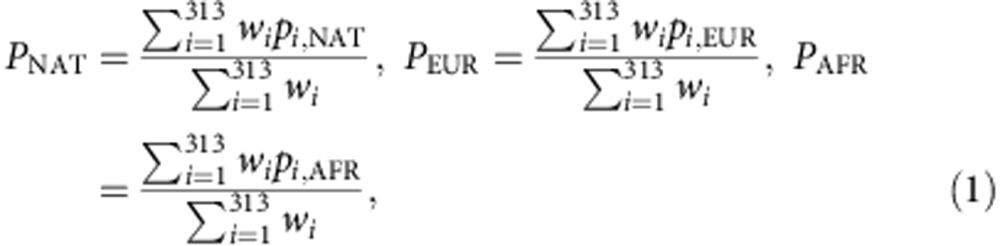


correspond to the Native-American, European and African ancestry proportion estimators for the Chileans.

### Consistency assessment of local-ancestry inference methods

To evaluate how consistent the local ancestry estimates are at every locus in each individual between both methods, LAMP-LD and RFMix, we count the number of loci in which both methods differ and look at specific patterns from loci with inconsistent assignments. Specifically, we perform correlation tests to assess whether SNPs in which both methods differ are also more common among SNPs showing larger differences in allele frequency at the ancestral populations or are within regions with recombination hotspots. We obtain the recombination rates from the European HapMap panel ( www.hapmap.org). The main reasons for using the EUR recombination map instead of a Latino map are the following: (i) even though recombination maps have been estimated from some Latino population, the European is the only one that has an accurate pedigree-based map; (ii) at a coarse scale the Latino recombination map correlates well with the EUR map (r2=0.95); (iii) Chileans have on average greater ancestry from Europe than that from Native America.

### Effect of the size of reference panels on global ancestry

To estimate the variability on the individuals’ global ancestry proportion estimators due to the size of the reference panels and to propose a minimal optimal size for each reference panel, we implement the following procedure. We fixed the tuning parameters for LAMP-LD and RFMIx and varied the number of haplotypes at each reference panel. For LAMP-LD we fixed the number of states (*S*=15) and the length of the window (*L*=75), and for RFMix we fix the number of generations since the first admixture event occurred (*G*=8).

We start with 10 haplotypes for each reference panel and increase this number by 10 until we obtain a minimum number that stabilizes the estimates of the population global ancestry proportion in the Chilean samples. We denote this number by *X*_0_. Starting from *X*_0_ haplotypes at each reference panel, we then increase the sample size solely at the Native-American panel until a stability criterion is achieved (we called this number *X*_Am_). Then, starting from *X*_Am_ haplotypes in the NAT population and *X*_0_ haplotypes in the EUR and AFR populations we increase the size of the EUR panel until the same stability criterion is achieved (we called this number *X*_E_). Last, we start with *X*_Am_ haplotypes in the NAT population, *X*_E_ haplotypes in the EUR population and *X*_0_ haplotypes in the AFR population and increase the number of haplotypes in the AFR panel until the same stability criterion is achieved (we called this number *X*_Af_). To avoid possible biases due to sample selection, and to be able to estimate the variability as a function of reference panel sizes, we repeat the procedure 10 times. The stability criterion that we consider was either the minimum median s.d. (in the cases of the NAT and AFR populations) or, for the EUR population, we use a criterion based on the median estimates of s.d. for every sample size. We identify a ‘kink’ in the plot such that 
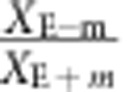
 is large for every *m* (see Fig. 8).

### LAI method’s sensitivity to the density of SNP panels

To determine a minimum number of SNPs with which we can obtain estimates of global ancestry proportions with low variability, we evaluate the performance of LAMP-LD as we vary the density of markers on chromosome 1.

We fix the reference panel to (176, 410, 458) haplotypes at the (NAT, EUR, AFR) population, respectively, and vary the number of SNPs selected randomly (*N*_snaps_ε{70, 80, 90, 100, 200,…, 1,000,…,10,000}). As we start with a small number of SNPs (that is, 70), we fix window length to *L*=10. To avoid bias due to sample selection, we repeat this procedure 10 times to obtain for every admixed individual an average and s.d. of global ancestry proportions at each SNP-density-size.

## Author contributions

S.E. conceived the project, S.E. and F.M. analysed the data, S.E. drafted the manuscript. F.I.M., G.M.R. and C.V. collaborated in manuscript writing, G.M.R. and C.V. designed the GWAS studies, obtained funding, enrolled participants and collected information on their geographic origins, performed/supervised array experiments. All authors reviewed and accepted the final version.

## Additional information

**Accession codes.** Genetic polymorphism data have been deposited in NCBI dbSNP database under accession code 1062069.

**How to cite this article:** Eyheramendy, S. *et al*. Genetic structure characterization of Chileans reflects historical immigration patterns. *Nat. Commun*. 6:6472 doi: 10.1038/ncomms7472 (2015).

## Supplementary Material

Supplementary Information Supplementary Figure 1 and Supplementary Tables 1-3

## Figures and Tables

**Table 1:** Sample collections.

Population	Group	Sample size	Source
CHL, Chile	CHL	313	This study
AYM, Aymara	NAT	25	60,61
QUE, Quechua	NAT	24	60,61
NAH, Nahua, Mixtec, Tlapanec	NAT	14	60,61
MAY, Maya	NAT	25	60,61
CEU, North Europe	EUR	85	1000GP
GBR, Britain	EUR	89	1000GP
IBS, Iberians from Spain	EUR	14	1000GP
SPN, Spain	EUR	625	POPRES
TSI, Toscani in Italy	EUR	98	1000GP
ASW, African Ancestry from Southwest United States	AFR	56	1000GP
LWK, Luhya in Webuye, Kenya	AFR	85	1000GP
YRI, Yoruba in Ibadan, Nigeria	AFR	88	1000GP

Summary table of the populations used in this study.

**Table 2:** Political regions.

Political region	Samples (total)	Population
Arica y Parinacota	0.6% (2)	1.1%
Tarapacá	1.6% (5)	1.8%
Antofagasta	0.6% (2)	3.4%
Atacama	1.0% (3)	1.6%
Coquimbo	1.3% (4)	4.2%
Valparaíso	9.3% (29)	10.3%
Metropolitana	39.6% (124)	40.3%
O’Higgins	1.9% (6)	5.2%
Maule	2.9% (9)	5.9%
Bio-bio	10.2% (32)	11.9%
Araucanía	8.3% (26)	5.7%
Los Ríos	8.3% (26)	2.2%
Los Lagos	6.7% (21)	4.9%
Aysén	2.9% (9)	0.6%
Magallanes	1.0% (3)	0.9%
Unknown	3.8% (12)	—

Political region names (first column), percentages and number of individuals in the sample (second column), and the percentages in the population (third column).
