# Supplementary material for: Genetic structure characterization of Chileans reflects historical immigration patterns
Source: Nat Commun. 2015 Mar 17;6:6472. doi: 10.1038/ncomms7472 (PMC4382693; doi:10.1038/ncomms7472)
Supplement: Supplementary Information — Supplementary Figure 1 and Supplementary Tables 1-3 [file ncomms7472-s1.pdf]

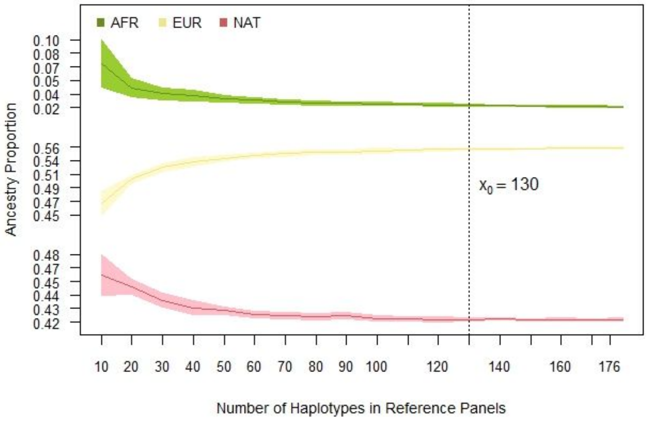

Supplementary Figure 1. Variation in ancestral component proportions as the number of haplotypes in reference panels grow. All 3 curves show certain asymptotic convergence. At $x_0 = 130$, both standard errors and mean values met an initial convergence criteria.

	Chr	SNP	GENE		Chr	SNP	GENE
1	4	rs41337747	RNF150	51	12	rs7311761	intergenic
2	2	rs41497646	LRP1B	52	2	rs260711	EDAR
3	7	rs6964752	IQCE	53	6	rs3103418	intergenic
4	16	rs8054781	intergenic	54	5	rs4145160	GRIA1
5	16	rs7404672	CIITA	55	1	rs10458591	intergenic
6	3	rs17008458	intergenic	56	3	rs7650582	ADAMTS9
7	2	rs6436396	intergenic	57	10	rs17130359	intergenic
8	3	rs1463390	MYLK	58	1	rs16854428	intergenic
9	4	rs10016699	intergenic	59	13	rs9571492	intergenic
10	3	rs10510511	intergenic	60	1	rs12025524	intergenic
11	2	rs260690	EDAR	61	12	rs7967620	SVOP
12	4	rs11725412	intergenic	62	10	rs2419834	intergenic
13	1	rs2025236	intergenic	63	2	rs1196702	LYPD6
14	3	rs6780694	intergenic	64	1	rs10458589	intergenic
15	4	rs973786	intergenic	65	3	rs1586861	intergenic
16	4	rs12649145	intergenic	66	2	rs905307	intergenic
17	4	rs12643176	intergenic	67	1	rs10458590	intergenic
18	4	rs4279220	intergenic	68	5	rs35407	SLC45A2
19	3	rs1344870	intergenic	69	2	rs3806510	KLHL29
20	4	rs12649142	intergenic	70	12	rs11065850	intergenic
21	1	rs6672027	intergenic	71	5	rs35389	SLC45A2
22	16	rs7189406	CIITA	72	11	rs12273080	TPCN2
23	1	rs6681719	LOC643355	73	4	rs1552398	intergenic
24	8	rs11778591	intergenic	74	4	rs17219642	intergenic
25	1	rs11240271	intergenic	75	2	rs3806508	KLHL29
26	3	rs1836953	intergenic	76	9	rs10116041	intergenic
27	1	rs6593907	intergenic	77	13	rs1409264	GPC5
28	3	rs1344869	intergenic	78	11	rs11038170	TSPAN18
29	10	rs11196359	intergenic	79	6	rs10498810	COL21A1
30	10	rs4261225	intergenic	80	10	rs17130385	upstream HABP2
31	22	rs1557553	intergenic	81	2	rs4852057	intergenic
32	14	rs41348245	intergenic	82	2	rs6746925	SH3RF3
33	4	rs7664927	ANK2	83	5	rs17746614	MIR4454
34	4	rs16994362	intergenic	84	6	rs1925188	COL21A1
35	10	rs3810947	CHAT and SLC18A3	85	2	rs7588387	SH3RF3
36	3	rs1605524	intergenic	86	2	rs4952584	inergenic
37	4	rs7664966	ANK2	87	2	rs260705	EDAR
38	2	rs4952644	intergenic	88	14	rs10139931	intergenic
39	2	rs3915716	intergenic	89	13	rs2762998	XPO4
40	10	rs2419830	intergenic	90	14	rs41320346	intergenic
41	15	rs12442144	RGMA	91	11	rs1079147	TSPAN18
42	10	rs7919248	intergenic	92	14	rs10142932	intergenic
43	16	rs3760114	intergenic	93	12	rs1163895	intergenic
44	15	rs17649077	RGMA	94	21	rs2837352	intergenic
45	2	rs13417715	HDAC4	95	12	rs12229055	CCDC38
46	10	rs4918821	intergenic	96	6	rs4593388	C6orf99
47	2	rs11124754	intergenic	97	12	rs11169212	intergenic
48	5	rs3811984	GRIA1	98	22	rs9626091	PARVB
49	11	rs174570	FADS2	99	2	rs11899009	intergenic
50	5	rs2042314	intergenic	100	13	rs636437	intergenic

Supplementary Table 1. List of top 100 SNPs with large frequency difference among populations

	< 0.1%	< 0.5%	< 1%	< 3%	< 6%
NAT	49	205	277	308	313
EUR	31	180	275	309	313
AFR	114	283	308	313	

Supplementary Table 2. Differences in global ancestry estimates across LAMP-LD and RFMix. The first column of the table corresponds to the number of individuals in the sample that differ in less than 0.1% of the ancestry assigned by the two methods on Native American, European and African ancestries respectively. The following columns are defined similarly.

LAMP-LD \ RFMix	NAT	EUR	AFR
NAT	35.74	8.31	0.28
EUR	7.33	45.37	0.72
AFR	0.29	0.72	1.23

Supplementary Table 3. Table of consistent/inconsistent assignments of local ancestry between LAMP-LD and RFMix. Entries in this table correspond to the percentages of ancestry assignments by LAMP-LD on the rows and RFMix on the columns.
